# An Automated Home-Cage System to Assess Learning and Performance of a Skilled Motor Task in a Mouse Model of Huntington’s Disease

**DOI:** 10.1523/ENEURO.0141-17.2017

**Published:** 2017-09-18

**Authors:** Cameron L. Woodard, Federico Bolaños, James D. Boyd, Gergely Silasi, Timothy H. Murphy, Lynn A. Raymond

**Affiliations:** 1 Department of Psychiatry, Kinsmen Laboratory of Neurological Research, University of British Columbia, Vancouver, British Columbia V6T 1Z3, Canada; 2 Djavad Mowafaghian Centre for Brain Health, University of British Columbia, Vancouver, British Columbia V6T 1Z3, Canada; 3 Graduate Program in Neuroscience, University of British Columbia, Vancouver, British Columbia V6T 1Z3, Canada; 4 Department of Cellular and Molecular Medicine, Faculty of Medicine, University of Ottawa, Ottawa, Ontario K1H 8M5, Canada

**Keywords:** home-cage, Huntington’s disease, kinematic analysis, motor learning, novel methods

## Abstract

Behavioral testing is a critical step in assessing the validity of rodent models of neurodegenerative disease, as well as evaluating the efficacy of pharmacological interventions. In models of Huntington’s disease (HD), a gradual progression of impairments is observed across ages, increasing the need for sensitive, high-throughput and longitudinal assessments. Recently, a number of automated systems have been developed to perform behavioral profiling of animals within their own home-cage, allowing for 24-h monitoring and minimizing experimenter interaction. However, as of yet, few of these have had functionality for the assessment of skilled motor learning, a relevant behavior for movement disorders such as HD. To address this, we assess a lever positioning task within the mouse home-cage. Animals first acquire a simple operant response, before moving to a second phase where they must learn to hold the lever for progressively longer in a rewarded position range. Testing with this paradigm has revealed the presence of distinct phenotypes in the YAC128 mouse model of HD at three early symptomatic time points. YAC128 mice at two months old, but not older, had a motor learning deficit when required to adapt their response to changes in task requirements. In contrast, six-month-old YAC128 mice had disruptions of normal circadian activity and displayed kinematic abnormalities during performance of the task, suggesting an impairment in motor control. This system holds promise for facilitating high throughput behavioral assessment of HD mouse models for preclinical therapeutic screening.

## Visual Abstract

**Figure:**
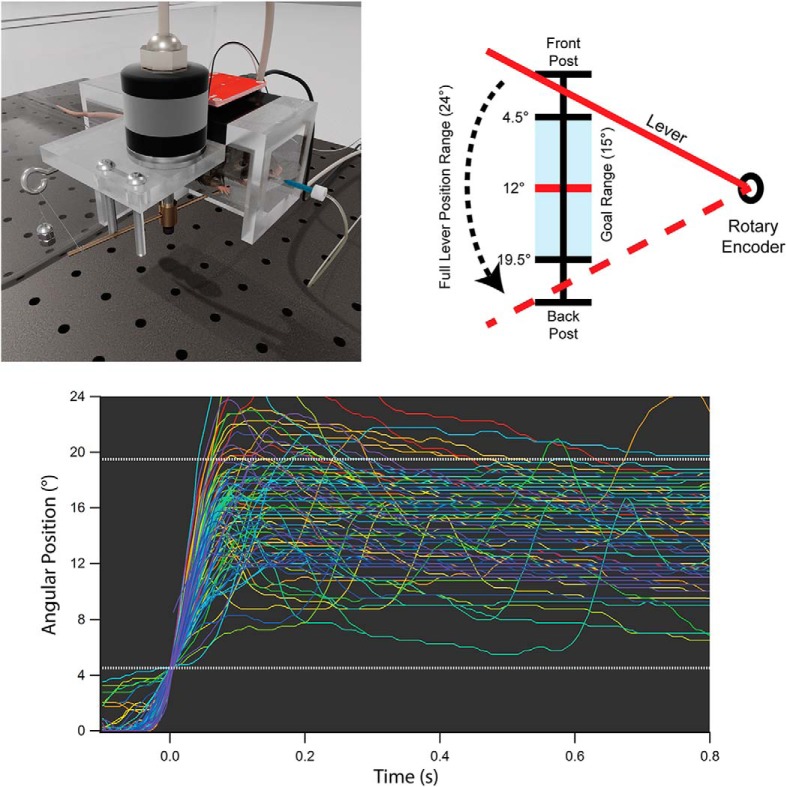

## Significance Statement

Difficulty with the learning and performance of skilled motor tasks is a common feature observed in many movement disorders, including Huntington’s disease (HD) and Parkinson’s disease. Modeling these characteristics is an important goal in our ongoing effort to understand the mechanisms by which these diseases progress, as well as in the search for prospective therapies. In this article, we use an automated behavioral testing system to assess learning and performance of a lever positioning task in a mouse model of HD, revealing several parallels with the human disease. We hope that this methodology will provide a more high-throughput platform for the behavioral screening of drugs that may help in the treatment of HD and similar diseases.

## Introduction

The past several decades have seen the widespread development and application of transgenic mouse models for the study of brain disorders. These animal models serve both to elucidate the underlying mechanisms of genetic disorders, as well as provide a platform for the pre-clinical screening of potential therapeutic interventions. Huntington’s disease (HD), an autosomal dominant genetic disorder, is one such disease that has benefited from genetic modeling in mice. HD is caused by a trinucleotide repeat expansion on the gene huntingtin (*HTT*; Huntington’s Disease Collaborative Research Group, 1993), and mutation carriers most often show a progressive deterioration in motor function starting in middle-age (Kirkwood et al., 2001). However, HD is not solely a movement disorder; cognitive decline is eventually observed in all patients, and deficits on certain cognitive and motor tasks can precede the onset of disease diagnosis by 10-15 years (Paulsen et al., 2008). In addition, psychiatric illness, primarily depression, is highly comorbid with both pre-symptomatic and clinical HD (Kirkwood et al., 2001; Julien et al., 2007).

To date, over 50 distinct mouse and rat models of HD have been developed (Pouladi et al., 2013; Menalled et al., 2014), and behavioral testing is a critical step in determining how closely aligned the animal’s phenotype is with human symptomatology (often referred to as the “face validity” of the model). The YAC128 mouse model expresses the human full-length *HTT* gene with 128 CAG repeats on a yeast artificial chromosome construct, and has been well established to show many of the behavioral and pathophysiological features of the human disease (Slow et al., 2003). These animals display motor and balance deficits, as well as cognitive impairments in learning, memory and strategy shifting starting as early as two months old (for review, see Abada and Ellenbroek, 2016). However, conflicting results have been reported concerning the time frame, severity and progression of some behavioral phenotypes, including motor learning (Van Raamsdonk et al., 2005; Menalled et al., 2009; Brooks et al., 2012a). Although differences in methodology and apparatus may be contributing to this variability in behavioral outcome measures (Mandillo et al., 2008), other factors likely include the variable expressivity of behavioral phenotypes between animals, and the testing of insufficiently large experimental groups.

To address these issues, importance must be placed on finding novel ways to assess behavior that reduce confounding factors and increase throughput. In recent years, several systems have become commercially available which increase the automation of behavioral testing and analysis by assessing animals within their home-cage (e.g., Intellicage; NewBehavior AG). These systems have the combined benefits of increasing the throughput of behavioral phenotyping, eliminating the subjectivity associated with manual scoring, increasing the length of the testing period and reducing the amount of animal-experimenter interaction (Hånell and Marklund, 2014). Two recent papers have applied these paradigms to great success in HD models, using computer vision techniques in combination with machine learning to develop a comprehensive behavioral profile for a set of HD animal models (Balci et al., 2013; Alexandrov et al., 2016).

Recently, interest has been growing in the development of automated systems for the assessment of skilled motor tasks (Fenrich et al., 2015; Sindhurakar et al., 2017). Although rotarod and other full body coordination tasks are sensitive in capturing one aspect of motor function and balance, they may not detect more subtle motor learning and movement kinematic phenotypes relevant to HD. To address this, we have employed a lever-positioning task that integrates into the animal’s home cage, and is accessible by group-housed mice full-time over several weeks of testing. This task is learned in a self-directed manner, and following initial acquisition of a simple operant response, task demands change dynamically to probe motor learning and behavioral flexibility. Additionally, this system collects kinematic measures of task performance, allowing for the measurement and detection of motor abnormalities. In the present article, we report our results from using this system for the assessment of several motor, circadian and cognitive phenotypes in the YAC128 model at two, four, and six months of age. We found that while two-month-old YAC128 mice had difficulties with adapting their motor behavior in response to changes in task demands, older mice did not show this problem. Conversely, alterations in the kinematics of task performance were evident in six-month-old mice, but not seen in the younger animals. Additionally, YAC128 mice across ages had circadian abnormalities in the distribution of their trials, and a greater number failed to acquire the task as compared to WT.

## Materials and Methods

### Animal housing, husbandry, and genotyping

A colony of heterozygous YAC128 mice on the FVB/N background (YAC128 line 53, RRID:MGI:3613525; Slow et al., 2003) was maintained by breeding with wildtype FVB/N animals. Animals were housed in cages of two to five male littermates on a 12/12 h light/dark cycle in a temperature- and humidity-controlled room. A total of 123 male WT and YAC128 animals were used for experiments, and all procedures were conducted in accordance with the Canadian Council on Animal Care and approved by the University of British Columbia Committee on Animal Care. Until the start of testing, animals were allowed *ad libitum* access to standard lab chow and water. Animal tissue was collected via ear clipping at weaning, and DNA extraction and PCR analysis were subsequently used to determine genotype, as previously described (Slow et al., 2003). As the experiments involved minimal experimenter interaction or handling of the mice, and no subjective analysis was performed, the experimenter was not blinded to genotype.

### RFID microchip implantation

Glass RFID capsules (Sparkfun, SEN-09416) were implanted into all animals before behavioral testing as described in Bolaños et al. (2017). Briefly, surgical plane was induced with 4% isofluorane in an induction chamber, followed by a switch to 1.5% isofluorane for maintenance. The thoracic torso was disinfected with betadine and a sterile injector (Fofia, ZS006) was used to penetrate the dermal layer and insert the RFID capsule below the nape of the neck. Buprenorphine was administered via subcutaneous injection (0.05 mg/kg) and animals were allowed to recover from anesthesia while being monitored for signs of pain. Animals were moved back to their home-cage and checked again 24 h later to ensure normal behavior and proper placement of microchips.

### Description of apparatus

**Figure 1.**
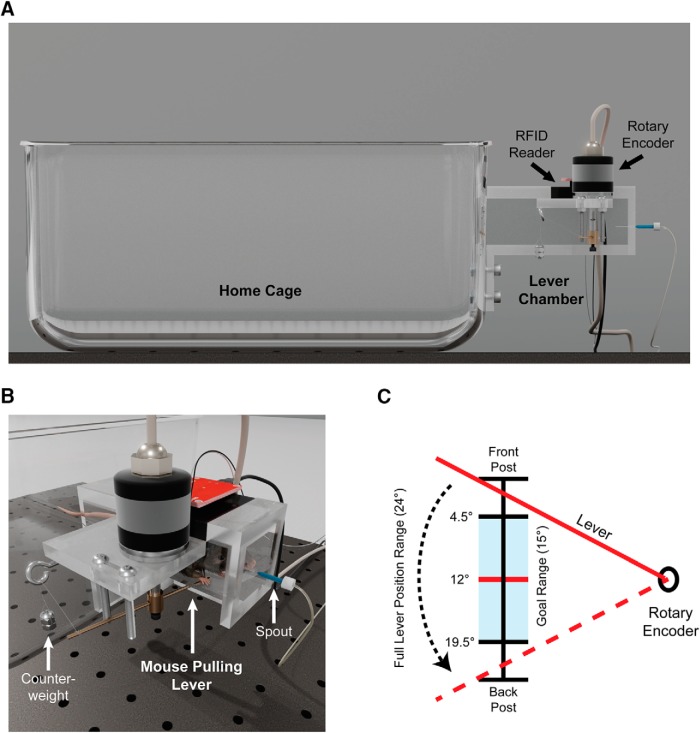
Apparatus for home-cage assessment of skilled motor learning. ***A***, A small opening on one side of the home-cage allows 24-h access to a chamber containing a metal lever and water spout. Microchipped animals are identified by an RFID reader on entrance into the chamber, allowing for individual tracking and assessment of group-housed animals. ***B***, The lever is restricted in its horizontal movement by two metal posts, and held in starting position by a small counterweight. In the first phase of testing, the mouse must pull the lever backwards 12° from its starting position to receive a water drop. ***C***, A top-down view of the lever position range. In the second phase of testing, the mouse must first pull the lever back to the center (red line), and then hold it within a central goal position range (shaded area) to receive a water drop. The length of time the lever must be held for changes dynamically based on the individual animal’s success rate.

All experiments were performed in a modified mouse home-cage (referred to herein as the “lever-cage”), with a custom designed Plexiglas rectangular prism chamber (2.5 × 2.5 × 9.5 cm) attached to one side, 6 cm from the floor of the cage (Fig. 1*A*,*B*). This chamber is closed on all sides except for an opening leading into the cage, and a second narrow opening of 3 cm along the bottom of the right wall at the end opposite the cage entrance. A cylindrical steel rod (2 mm thick) extends through this opening ∼1 cm into the chamber. This rod is movable on a horizontal axis, restricted to a range of 24° by two metal posts, and held in its “starting” position by a 1.5-g counterweight (Fig. 1*C*). The lever is also attached to a rotary encoder (Phidgets, ISC3004) to measure and record all movements. On the far wall of the chamber adjacent to the lever, a spout (blunted 21-G needle) dispenses water drops, and is attached to a computer-controlled valve and water supply (Murphy et al., 2016). An RFID antenna and reader (Sparkfun) is inset into the ceiling of the chamber to individually identify microchipped animals. A description of the water delivery system and RFID tag electronics and software can be found in Murphy et al. (2016) and Bolaños et al. (2017). All components are controlled by customized software running on a Raspberry Pi single-board computer. Mice are provided with free access to chow and have standard environmental enrichment within the cage (bedding, hut, PVC tube).

### Behavioral testing

Animals were transferred to an animal facility following RFID microchipping and allowed to recover and habituate for a minimum of 5 d before the start of testing. Naïve animals began the testing protocol at 60, 120, or 180 d old (±5 d). Animals were tested alongside their littermates in mixed genotype groups of two to five animals per cage.

**Figure 2.**
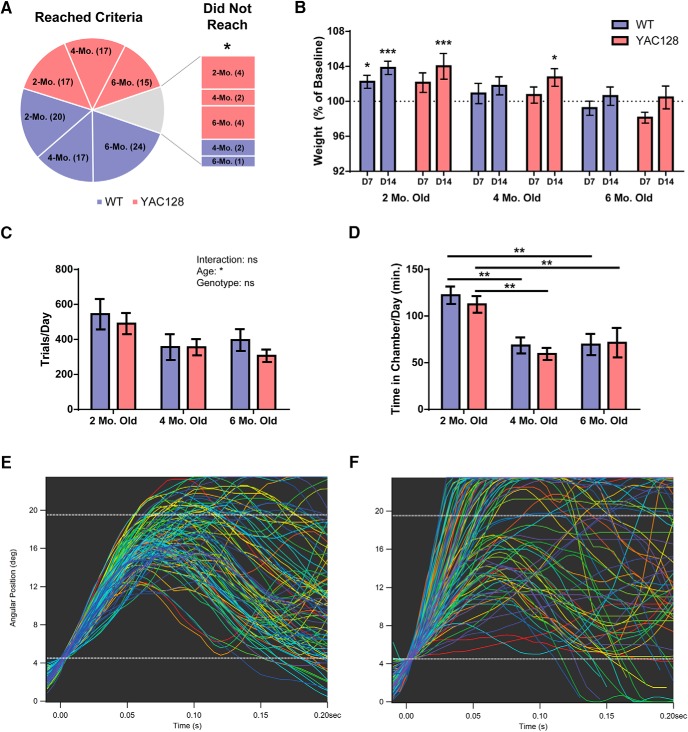
Acquisition and performance of lever-pull task in phase 1. ***A***, Number of animals to reach the performance criteria of 200 trials performed in phase 1. An overall lower proportion of YAC128 animals acquired the task as assessed by this cutoff. ***B***, Average weight over the course of testing as a percentage of baseline. Although six-month-old animals remained at their baseline weight, two-month-old WT and YAC128 animals and four-month-old YAC128 animals gained weight over 14 d in the lever-cage (asterisks indicate significant increase as compared to baseline weight). ***C***, No significant differences between WT and YAC128 were seen in the number of trials performed per day; however, animals in both genotypes performed less daily trials with increasing age. ***D***, Time spent in the chamber per day was also not significantly different between genotypes; however, both WT and YAC128 animals were much higher on this measure at two months old than at other ages. ***E***, ***F***, Sample lever traces from two four-month-old animals (WT and YAC128, respectively) in phase 1. Each line represents one trial. Numbers of animals (WT/YAC128) used for weight, trial frequency, and time in chamber analysis are *n* = 17/13 at two months old, *n* = 14/16 at four months old, and *n* = 18/12 at six months old. All data are presented as mean ± SEM. ns = not significant; *p < 0.5; **p < 0.01; ***p < 0.001; ****p < 0.0001.

In the initial phase of testing (phase 1), naïve animals were introduced to the lever-cage and allowed to explore and discover the chamber. Entrance into the chamber by an animal triggered the RFID reader, resulting in the delivery of a single water drop (5 µl) from the reward spout, up to a maximum of 200 drops per day. Additional water drops (10 µl) could be obtained on a continuous reinforcement schedule by pulling the lever backwards past the center of its movable range (12° from starting position). The chamber was accessible to animals 24 h/d, and the timing of each entrance, exit, and trial was automatically collected and saved. Additionally, the position trace of the lever during each trial was recorded for kinematic analysis. This initial testing period lasted from 3-8 d, and animals were not disturbed once introduced to the cage except for semi-weekly weighing and bedding changes. Animals that did not perform >200 trials during this initial acquisition period were removed from the cage and not used for further testing (Fig. 2*A*; Table 1). Additionally, one six-month-old YAC128 mouse lost >15% body weight during this initial testing period and was removed from the cage and not used for analysis (Table 1).

**Table 1.** Animals excluded from analysis

	Animals initially available for testing	Did not reach criteria in phase 1	Did not reach maximum hold duration	Cage crash or malfunction	Excessive weight loss
Two months old	20 WT/21 YAC128	0 WT/4 YAC128	0 WT/1 YAC128	7 WT/5 YAC128	0 WT/0 YAC128
Four months old	19 WT/19 YAC128	2 WT/2 YAC128	2 WT/1 YAC128	5 WT/3 YAC128	0 WT/0 YAC128
Six months old	25 WT/19 YAC128	1 WT/4 YAC128	1 WT/0 YAC128	9 WT/5 YAC128	0 WT/1 YAC128

Following acquisition of the basic lever-pull task, animals were moved to a second phase (phase 2) where the criteria to receive a water drop changed. As before, the animal was required to displace the lever to the center of its movable range. However instead of immediately receiving a drop, the lever then had to be held in a central “goal range” (between 4.5° and 19.5° from start position) for a prescribed length of time before a drop was dispensed (Fig. 1*C*). If the lever was not held in the range for the required duration, a failed trial was recorded and no water was dispensed. Initially, this hold duration was set to a minimum of 100 ms for all animals, but this could increase based on the animal’s performance of the task. Every 25 trials, a ratio of successful to failed trials was calculated for that animal; if the animal had a >75% success rate over this block, then the required hold duration increased by 100 ms, to a maximum of 800 ms (an end goal that the large majority of animals were able to achieve in pilot experiments). If the animal was <10% successful, the required hold duration decreased by 100 ms. Otherwise, the hold duration remained the same for the subsequent block of trials. After 7 d in phase 2, animals were removed from the lever-cage and returned to their regular home-cage. Only five animals did not reach the maximum hold duration within the 7 d (Table 1).

### Data analysis and statistics

All task performance data were automatically recorded into text files by the lever-cage software and were subsequently extracted and analyzed by customized scripts using IGOR Pro (Wavemetrics, RRID:SCR_000325). For analysis of kinematic measures in phase 2, all successful trials at the maximum hold duration (800 ms) were averaged to determine mean maximum displacement, speed, and slope of trajectory for each animal. Only animals with a minimum of 200 eligible trials before the end of testing were used to obtain a representative average and reduce the effect of intertrial variability. During the course of testing, some animals were excluded from analysis at intermediary stages because of system crashes that resulted in interruption of task access, and several other animals were excluded because of faulty data collection or program errors that led to nonstandard task advancement. Numbers of animals used for each analysis are indicated in figure legends, and numbers of animals excluded, with reasons why, are summarized in Table 1.

All statistical analyses were performed using GraphPad Prism 6.01 (GraphPad Software, RRID:SCR_002798). For most datasets, regular or repeated measures two-way ANOVA (as appropriate) with Bonferroni *post hoc* tests was used for statistical analysis of main/interaction effects. For the analysis of trials performed per day, a log transformation was used to normalize the data to allow for the use of two-way ANOVA, as several of the groups had a strong right skew in their distribution. For the analysis of time spent in the lever chamber, a significantly non-Gaussian distribution was seen in many of the groups, limiting the use of two-way ANOVA. Paired comparisons between genotypes at each age group using either Student’s *t* tests or Mann-Whitney tests was performed, in addition to Kruskal-Wallis tests with Dunn’s *post hoc* tests to analyze age effects in each genotype group. Fisher’s exact test was used to compare the proportion of animals that reached criteria in phase 1 and reached the maximum hold duration in phase 2. A full summary of statistical results can be found in Table 2.

**Table 2.** Statistical table of all analyses

	Data structure	Type of test	Test values and power
Fig. 2*A*	N/A	Fisher’s exact test	*p* = 0.0386
Fig. 2*B*, two months	All but one group normally distributed (D7 WT)	Repeated measures two-way ANOVA with Bonferroni *post hoc* tests	Days in cage: *F*_(2,56)_ = 20.11, *p* < 0.0001; genotype: *F*_(1,28)_ = 0.0007, *p* = 0.9798; interaction: *F*_(2,56)_ = 0.0260, *p* = 0.9743
Fig. 2*B*, four months	All groups normally distributed	Repeated measures two-way ANOVA with Bonferroni *post hoc* tests	Days in cage: *F*_(2,56)_ = 6.050, *p* = 0.0042; genotype: *F*_(1,28)_ = 0.08113, *p* = 0.7779; interaction: *F*_(2,56)_ = 0.4340, *p* = 0.6501
Fig. 2*B*, six months	All groups normally distributed	Repeated measures two-way ANOVA with Bonferroni *post hoc* tests	Days in cage: *F*_(2,56)_ = 3.936, *p* = 0.0252; genotype: *F*_(1,28)_ = 0.2267, *p* = 0.6376; interaction: *F*_(2,56)_ = 0.3738, *p* = 0.6898
Fig. 2*C* (log transform)	All but one group (six-month YAC128) normally distributed, equal variances	Two-way ANOVA	Age: *F*_(2,84)_ = 4.803, *p* = 0.0106; genotype: *F*_(1,84)_ = 0.1089, *p* = 0.7422; interaction: *F*_(2,84)_ = 0.5332, *p* = 0.5887
Fig. 2*D*, two months	Normal distribution, equal variances	Student’s *t* test	*t*_(28)_ = 0.7433, *p* = 0.4635
Fig. 2*D*, four months	Non-normal distribution	Mann-Whitney test	*U* = 94, *p* = 0.4659
Fig. 2*D*, six months	Non-normal distribution	Mann-Whitney test	*U* = 107, *p* = 0.9665
Fig. 2*D*, WT	Non-normal distribution	Kruskal-Wallis Test with Dunn’s *post hoc* tests	*H* = 15.22, *p* = 0.0005
Fig. 2*D*, YAC128	Non-normal distribution	Kruskal-Wallis Test with Dunn’s *post hoc* tests	*H* = 13.50, *p* = 0.0012
Fig. 3*B*	Groups normally distributed, equal variances	Two-way ANOVA	Age: *F*_(2,84)_ = 2.945, *p* = 0.0580; genotype: *F*_(1,84)_ = 4.772, *p* = 0.0317; interaction: *F*_(2,84)_ = 0.2492, *p* = 0.7800
Fig. 3*C*	Groups normally distributed	Repeated measures two-way ANOVA	Hour of day: *F*_(23,644)_ = 86.51, *p* < 0.0001; genotype: *F*_(1,28)_ = -0.3218, *p* > 0.9999; interaction: *F*_(23,644)_ = 0.7632, *p* = 0.7788
Fig. 3*D*	Groups normally distributed	Repeated measures two-way ANOVA with Bonferroni *post hoc* tests	Hour of day: *F*_(23,598)_ = 56.36, *p* < 0.0001; genotype: *F*_(1,26)_ = 0.0, *p* > 0.9999; interaction: *F*_(23,598)_ = 2.296, *p* = 0.0006
Fig. 3*E*	Groups normally distributed	Repeated measures two-way ANOVA with Bonferroni *post hoc* tests	Hour of day: *F*_(23,644)_ = 43.87, *p* < 0.0001; genotype: *F*_(1,28)_ = 0.8750, *p* = 0.3576; interaction: *F*_(23,644)_ = 1.911, *p* = 0.0066
Fig. 4*A*	Groups normally distributed	Repeated measures two-way ANOVA with Bonferroni *post hoc* tests	Trial number: *F*_(20,500)_ = 70.42, *p* < 0.0001; genotype: *F*_(1, 25)_ = 6.367, *p* = 0.0184; interaction: *F*_(20,500)_ = 5.321, *p* < 0.0001
Fig. 4*B*	Groups normally distributed	Repeated measures two-way ANOVA	Trial number: *F*_(20,460)_ = 115.8, *p* < 0.0001; genotype: *F*_(1,23)_ = 0.02924, *p* = 0.8657; interaction: *F*_(20,460)_ = 0.6740, *p* = 0.8528
Fig. 4*C*	Groups normally distributed	Repeated measures two-way ANOVA	Trial number: *F*_(20,520)_ = 115.7, *p* < 0.0001; genotype: *F*_(1,26)_ = 0.2737, *p* = 0.6053; interaction: *F*_(20,520)_ = 1.336, *p* = 0.1497
Fig. 4*D*	N/A	Fisher’s exact test	*p* = 0.7292
Fig. 4*E*	Groups normally distributed, equal variances	Two-way ANOVA	Age: *F*_(2,74)_ = 2.753, *p* = 0.0703; genotype: *F*_(1,74)_ = 2.002, *p* = 0.1613; interaction: *F*_(2,74)_ = 1.504, *p* = 0.2290
Fig. 5*E*	Groups normally distributed, equal variances	Two-way ANOVA with Bonferroni *post hoc* tests	Age: *F*_(2,64)_ = 3.193, *p* = 0.0477; genotype: *F*_(1,64)_ = 2.798, *p* = 0.0993; interaction: *F*_(2,64)_ = 2.522, *p* = 0.0883
Fig. 5*F*	All but one group (two-month YAC128) normally distributed, equal variances	Two-way ANOVA with Bonferroni *post hoc* tests	Age: *F*_(2,64)_ = 0.8329, *p* = 0.4395; genotype: *F*_(1,64)_ = 3.837, *p* = 0.0545; interaction: *F*_(2,64)_ = 6.309, *p* = 0.0032
Fig. 5*G*	Groups normally distributed, equal variances	Two-way ANOVA with Bonferroni *post hoc* tests	Age: *F*_(2,64)_ = 1.188, *p* = 0.3113; genotype: *F*_(1,64)_ = 2.193, *p* = 0.1435; interaction: *F*_(2,64)_ = 3.381, *p* = 0.0402

## Results

### WT and YAC128 mice rapidly acquire the task

The large majority of WT and YAC128 animals (∼90%) tested in all age groups successfully acquired the basic lever pulling task, and reached the performance criteria of 200 trials in phase 1. There was no difference in the proportion of animals that acquired the task between age groups or between genotypes within each age group, but there was an overall greater proportion of YAC128 animals that failed to reach the performance criteria within phase 1 as compared to WT (*p =* 0.0386; Fig. 2*A*). During the first few days of testing, some animals (especially at six months old) dropped in weight in response to the removal of *ad libitum* water. However, all but one animal recovered to within 10% of baseline weight after one week, and two-month-old WT mice gained weight over this period (*p =* 0.0485). Two-month-old mice, as well as four-month-old YAC128 mice, gained weight overall by the end of testing (two-month WT: *p =* 0.0001; two-month YAC128: *p =* 0.0004; four-month YAC128: *p =* 0.0211), whereas four-month-old WTs and six-month-old mice showed no change (Fig. 2*B*).

A substantial amount of interanimal variability was observed in the frequency of task performance among WT and YAC128 mice, with mice typically performing an average of 300-500 trials per day (Fig. 2*C*). An overall age effect was seen on trial frequency (*p =* 0.0106), with younger mice tending to have more trials per day, but no genotype differences were observed. A significant age effect was also seen in the amount of time spent in the testing chamber per day for both WT (*p =* 0.0005) and YAC128 (*p =* 0.0012) mice, with two-month-old animals higher on this measure than older animals (Fig. 2*D*). While some mice developed a relatively consistent strategy by the fifth day of testing, others were more variable in their performance, although no consistent genotype differences were observed (Fig. 2*E*,*F*).

### YAC128 mice display circadian abnormalities

**Figure 3.**
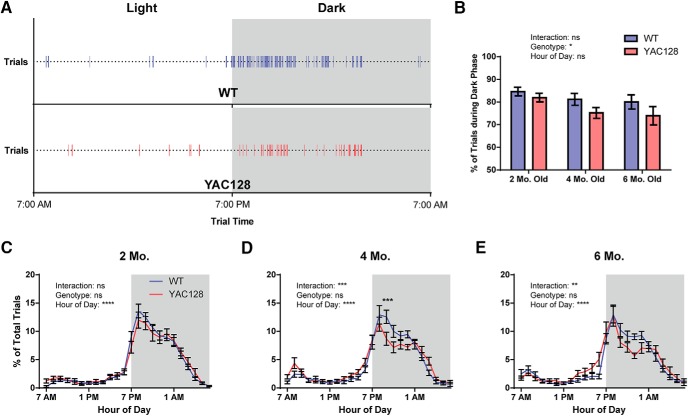
Distribution of trials throughout the light/dark cycle. ***A***, Raster plots show the distribution of trials through the day for representative four-month-old WT and YAC128 animals on the fifth day of testing (each line represents one trial). ***B***, The average percentage of all trials performed during the dark phase of testing was significantly higher in WT than in YAC128 mice, suggesting a disruption of normal circadian rhythms in these animals. ***C-E***, Trials were split into 1-h bins for each animal, and the percentage of trials occurring in each bin was calculated and graphed for two-, four-, and six-month-old age groups. A significant interaction between genotype and the hour of day was observed for four- and six-month-old, but not two-month-old, animals. Numbers of animals (WT/YAC128) used for analysis are *n* = 17/13 at two months old, *n* = 14/16 at four months old, and *n* = 18/12 at six months old. All data are presented as mean ± SEM. ns = not significant; *p < 0.5; **p < 0.01; ***p < 0.001; ****p < 0.0001.

Performance of the task was distributed throughout the day for individual animals, but an increase in activity was almost always observed during the first 6 h of the dark phase (Fig. 3*A*). Interestingly, when the overall proportion of light versus dark phase trials was analyzed, YAC128 mice were found to have significantly higher light phase activity than WT mice overall (genotype: *p =* 0.0317; Fig. 3*B*). To more closely examine this, we binned each animal’s trials by hour of day, and analyzed the average trial distribution for WT and YAC128 mice. While two-month-old YAC128 animals had no circadian abnormalities, there was a strong interaction between genotype and the timing of trials throughout the day in four-month-old (*p =* 0.0006) and six-month-old mice (*p =* 0.0066; Fig. 3*C-E*). YAC128 mice at these ages tended to increase their trial performance in the last 3 h of the light phase, and then drop steeply 2 h after the start of the dark phase, whereas WT mice maintained a higher trial performance rate through the first 6 h of the dark phase.

### Two-month-old YAC128 mice are impaired at adapting their motor response to changes in task demands

In phase 2, animals were required to hold the lever for progressively longer within a designated position range to receive water rewards. The way in which they progressed was based on their success rate at the current required hold duration, such that if over 75% of their trials in a 25-trial bin were held for the required length of time, the hold duration increased incrementally by 100 ms. Animals that were more successful at adapting to these changing demands had a higher success rate and consequently a faster progression to the maximum hold duration (800 ms). Conversely, animals that continued to perform their trials as in phase 1 did not advance.

**Figure 4.**
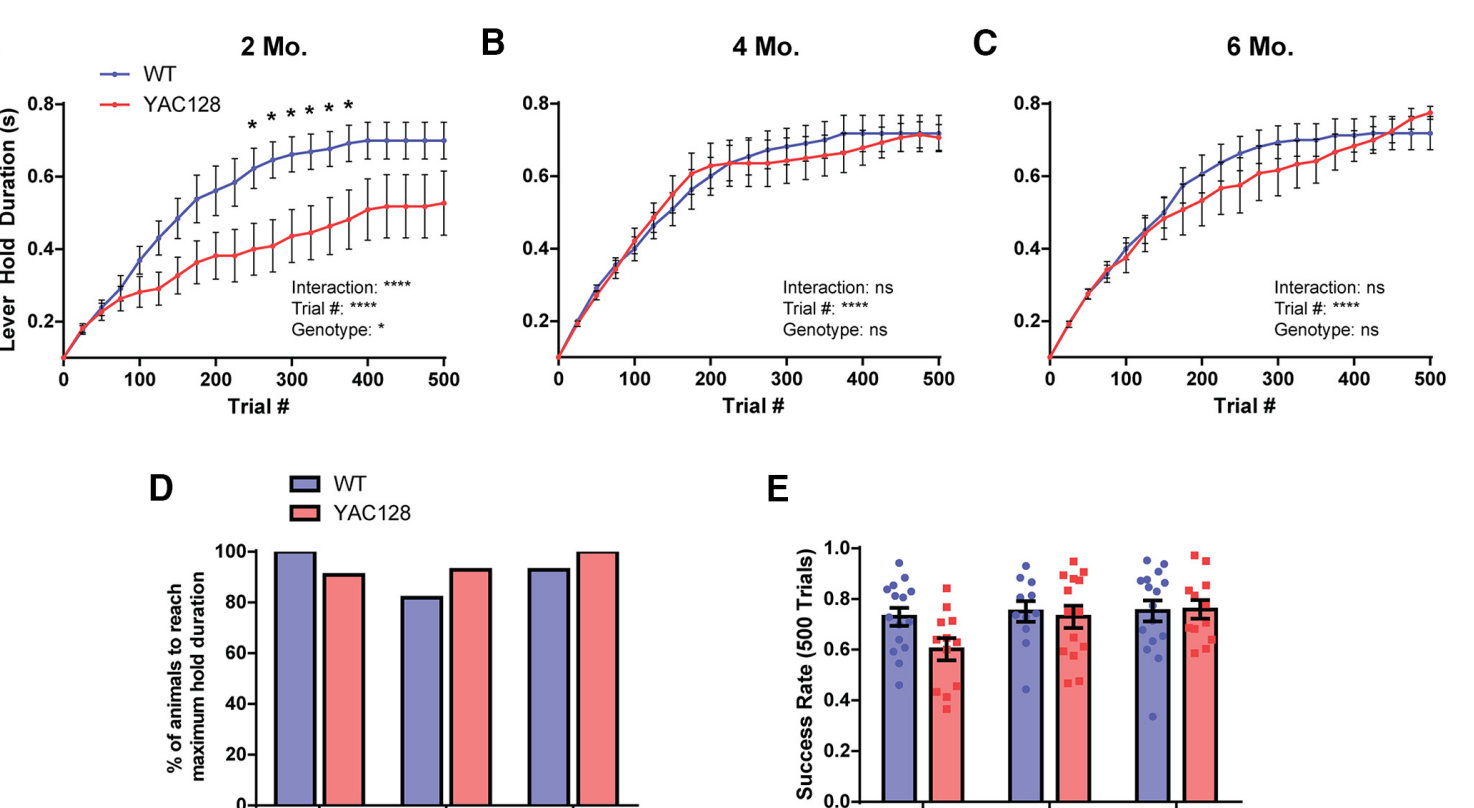
Performance of the task during phase 2. ***A-C***, Progression to the maximum required hold duration over the first 500 trials of phase 2 is plotted for two-, four-, and six-month-old age groups. At the end of each 25-trial bin, success rate was calculated over these trials to determine whether the animal met the threshold for their required hold duration to increase. Data are plotted as the required lever hold duration reached at the end of each 25-trial bin. YAC128 mice at two months old, but not other ages, had a significantly slower progression over the first 500 trials as compared to WT controls. ***D***. The majority of animals reached the maximum hold duration within one week, and no significant differences were observed between genotypes. ***E***, Success rate of animals over the first 500 trials of phase 2 is plotted for each age group. Two-month-old YAC128 animals had the lowest average success rate over this period, although no significant main or interaction effects were found. Numbers of animals (WT/YAC128) used for analysis are *n* = 15/12 at two months old, *n* = 11/14 at four months old, and *n* = 16/12 at six months old. All data are presented as mean ± SEM. ns = not significant; *p < 0.5; **p < 0.01; ***p < 0.001; ****p < 0.0001.

While four- and six-month-old YAC128 animals showed an equivalent progression through the task to their WT counterparts, two-month-old YAC128 mice showed a markedly slower progression, remaining at a lower required hold duration for longer on average before advancing (interaction: *p* < 0.0001; genotype: *p =* 0.0184; Fig. 4*A-C*). However, despite this slower progression, there were no genotype differences in the percentage of animals that eventually reached the maximum hold duration (Fig. 4*D*), suggesting that this was not a problem with meeting the physical demands of the task. This deficit in two-month-old YAC128 animals is also reflected in the overall success rate of these animals over the first 500 pulls of phase 2 (Fig. 4*E*). This group had a lower average success rate as compared to all other WT and YAC128 groups, although this difference was not significant (*p =* 0.0905).

### Six-month-old YAC128 mice have kinematic abnormalities when required to hold the lever for longer

**Figure 5.**
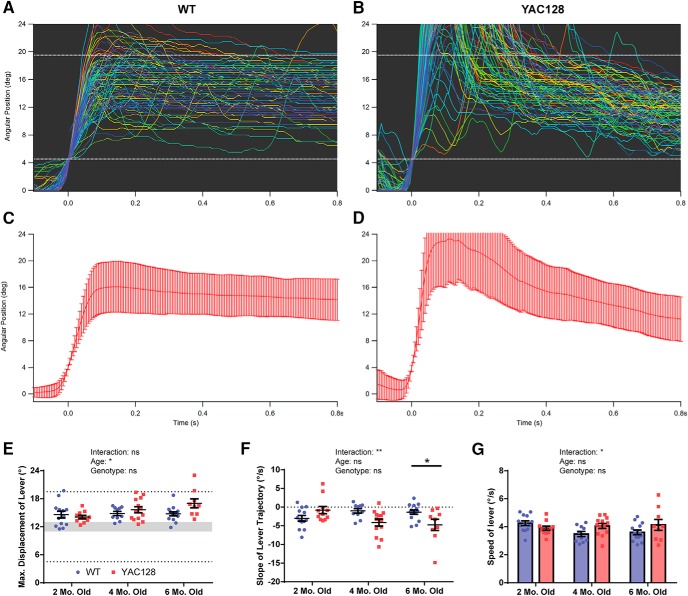
Kinematic measures of lever-pull trials at maximum hold duration. ***A***, ***B***, Lever position traces of 100 successful trials are shown for representative six-month-old WT and YAC128 mice who reached the maximum required lever hold duration. A tendency to overshoot the goal zone (dotted white lines) is seen in this YAC128 animal. ***C***, ***D***, Averaged lever position traces for the same two animals (error bars represent SD). ***E***, Average maximum displacement of the lever for all trials at the 800-ms hold duration is shown for WT and YAC128 animals. The shaded region represents the point at which a trial is initiated when pulled backwards (12 ± 1° from starting position), and the dotted lines represent the range it must be held within to receive a reward. A significant age effect was found, but not a significant genotype or interaction effect. ***F***, The average slope of the lever position trace from 200 to 800 ms after trial initiation was also calculated. An interaction between age and genotype was observed, and six-month-old YAC128 animals had a larger negative slope on average, indicating a progressive release of their hold on the lever. ***G***, The average speed of the lever over all trials at maximum hold duration. Although a significant interaction effect was seen, *post hoc* testing found no genotype differences in any of the age groups. Numbers of animals (WT/YAC128) used for analysis are *n* = 13/11 at two months old, *n* = 10/13 at four months old, and *n* = 14/9 at six months old. All data are presented as mean ± SEM except where indicated. ns = not significant; *p < 0.5; **p < 0.01; ***p < 0.001; ****p < 0.0001.

The change in performance of the task from phase 1 can be seen when looking at lever position traces of trials from WT and YAC128 animals that have reached the maximum hold duration (Fig. 5*A*,*B*). Instead of rapidly pulling back and then releasing, animals held the lever within the goal range for the designated amount of time, as was required to receive a reward. However, the strategy used to achieve this goal differed between WT and YAC128, specifically in the six-month-old group. Analysis of averaged traces revealed that WT animals at this age typically displace the lever to a point just past the center of the goal range, and hold it steady within this range until the end of the trial (Fig. 5*C*). In contrast, many six-month-old YAC128 mice pull the lever straight through the goal range, and then slowly allow it to return to its start position (Fig. 5*D*).

To quantify this, we took averages of several kinematic measures for each animal’s successful trials at the maximum required hold duration. The first of these was the maximum displacement of the lever from its starting position (i.e. the distance the lever is pulled backwards). A larger average maximum displacement was seen with increasing age (*p =* 0.0477) with this effect largely driven by an age-related increase in the YAC128 mice (Fig. 5*E*). As we did not observe a significant genotype or interaction effect overall, *post hoc* comparisons could not be performed between WT and YAC128 mice in any age group. However, a separate unpaired *t* test found a significant increase in maximum lever displacement in the six-month-old YAC128 mice (*t*_(21)_ = 2.405, *p =* 0.0255). During the subsequent 800-ms lever hold period, six-month-old animals had a greater negative slope of their lever trajectory on average (*p =* 0.0330), reflecting the progressive release of the lever during task performance (Fig. 5*F*). An interaction between age and genotype was also found in the average speed of the lever during each trial (*p =* 0.0402) due to a WT-specific decrease in this measure across ages, however this was not significantly different in any individual age group (Fig. 5*G*).

## Discussion

We present a fully automated home-cage methodology for investigating motor learning and movement kinematics in a mouse model of HD. We found that YAC128 HD mice display several distinct circadian, cognitive and motor abnormalities at different time points, although interestingly, some of these deficits did not progress with age as expected.

The first of these observed differences was that a larger proportion of YAC128 animals failed to reach the task performance criteria in our first phase of testing. One possibility is that YAC128 mice have a motor control deficit, and this performance failure reflects an inability to move the lever properly. However, many of these “noncriteria” animals initially performed trials but then quickly stopped, suggesting that physical ability to perform the task was not impaired. Additionally, this was not a progressive phenotype as might be expected if this was a motor control problem, as an equal number of animals at two and six months old failed to acquire the task. A second possibility is that this genotype difference may be due to a failure to learn the association between the lever response and water reward. Several papers have reported operant learning deficits in both YAC128 (Brooks et al., 2012b) and knock-in mouse models of HD (Trueman et al., 2007, 2008; Yhnell et al., 2016), supporting this first possibility. However, the majority of these deficits were related to the accuracy and reaction time of the HD animals. Significantly lower levels of task acquisition were only seen with a more difficult delayed matching to position task in HdhQ111 mice (Yhnell et al., 2016).

A final possibility is that these animals were capable of performing the response and learning the response-outcome contingency, but had reduced motivation to work for access to water. Animals tested in the lever-cage received a minimal amount of water (∼1 ml/d) simply by entering the chamber. However, this is much less than the ∼3 ml/d that FVB/N and YAC128 mice consume when allowed *ad libitum* water access (Pouladi et al., 2009), and is equivalent to what is typically given on a water restriction protocol (Guo et al., 2014). A depressive phenotype has previously been reported in YAC128 animals when tested on forced swim and sucrose preference tests (Pouladi et al., 2009, 2012), and so the failure of some animals to perform the task could be another reflection of these affective changes. This would be supported by the lack of age-related effects on this measure, as depressive and anhedonic phenotypes were not found to be progressive in these previous reports. Additionally, apathy, lack of motivation and depression are commonly reported in HD patients, and can occur long before the onset of motor symptoms (Kirkwood et al., 2001; Paulsen et al., 2001).

Weight fluctuations were observed in some animals during the first week of testing, especially in the six-month-old group. However, all animals (with the exception of one six-month-old YAC128 mouse) adapted to the restriction in water access and returned to within 5% of baseline weight by the end of testing (at minimum), suggesting that the change in *ad libitum* water access was well tolerated. The observed weight loss in older animals may be a reflection of this group having the highest baseline weight, and consequently highest dietary requirements for weight maintenance. In contrast, two-month-old mice, and four-month-old YAC128 mice, continued to grow during the testing period. Younger animals also tended to perform more trials, and consequently received more water, in comparison to older animals. As two-month-old mice have a higher growth rate as compared to older animals, the increased task performance observed at this age may reflect a higher level of motivation for water as compared to the older groups (www.jax.org/jax-mice-and-services/strain-data-sheet-pages/body-weight-chart-001800). While we have employed periodic manual weighing, it is notable that similar home-cages can be adapted to automated weighing and handling of up to 10 animals (Noorshams et al., 2017).

Abnormalities were also observed in YAC128 mice in the distribution of lever-pull trials over the course of the day. YAC128 mice performed more of their trials during the light phase overall, and subdividing trials into 1-h bins revealed distinct circadian irregularities specifically in the four- and six-month-old mice. While WT mice at these time points tended to have a very low percentage of their trials in the hours leading up to the start of the dark phase, YAC128 mice began to increase their performance of the task 3-4 h before this point. Furthermore, WT animals maintained a high performance rate over the first 6 h of the dark phase, whereas YAC128 mice began to decrease in their performance rate in the third hour. Circadian disruptions have been reported in patients with HD (Morton et al., 2005), as well as in several mouse models of HD, including R6/2, BACHD, and Q175 (Morton et al., 2005; Kudo et al., 2011; Oakeshott et al., 2011; Loh et al., 2013). However, similar circadian abnormalities have not previously been reported for YAC128 mice. Similarly to results published on other genetic lines, this is a progressive deficit and was not observed in the two-month-old animals. Although our task does not give a direct measure of overall activity level or locomotion, trial distribution through the day seems to provide a good proxy measure for this, and further confirms the disruptions observed in other genetic models.

In the second phase of testing, the success requirements of the task progressively changed, and animals were required to modify their motor response. The majority of animals were able to deal with these changes in task requirements and progressed quickly to the maximum required lever hold duration. However, two-month-old YAC128 mice had a significantly slower average progression through the stages of the task as compared to WTs. This was not due to difficulties with the physical demands of the task, as these two-month-old animals showed no kinematic abnormalities and a similar percentage of them reached the maximum hold duration. Rather, this deficit seems to reflect a persistence in using the previously learned strategy instead of adapting their behavior to meet the new requirements. The observation of a motor learning deficit is not surprising in itself, as YAC128 mice as young as two months old have previously been found to have slower learning on a fixed speed rotarod task (Van Raamsdonk et al., 2005). A mild reversal learning deficit was also seen at two months old in the water T-maze, with more robust effects seen in animals at 8.5 months old and older (Van Raamsdonk et al., 2005; Brooks et al., 2012c), and our results could also be a reflection of impaired behavioral flexibility. However, what is more surprising is that the four- and six-month-old YAC128 mice did not show a similar impairment. As no differences were seen between the WT animals at different time points, this seems to reflect an early transient deficit of YAC128 mice at this age.

Several other phenotypes reported in young YAC128 mice have been seen to normalize to WT levels at later time points. For example, YAC128 animals display an early hyperkinetic phenotype in open field testing at three months old, before later decreasing in their open field activity to WT levels by six months old (Slow et al., 2003). At a physiologic level, an early increase in spontaneous EPSCs has been reported in dopamine D1 receptor-expressing medium spiny neurons (MSNs) of YAC128 mice at 1.5 months of age; however, this is reduced to WT levels in six-month-old animals (André et al., 2011). Furthermore, modulation of spontaneous activity by D1 receptor activation was found to be lost in acute slices from YAC128 mice at 1.5 months old, but restored at six months old (André et al., 2011). D1 receptor function in direct pathway MSNs is an important regulator of synaptic plasticity (Kreitzer and Malenka, 2008), and it is possible that the motor learning deficit we observe is linked to overactivation and loss of synaptic plasticity at these striatal inputs. Another factor that may be contributing to this early and transient behavioral phenotype is changes in the activity and expression of the adenosine A2a receptor. An increased density of this receptor is seen in very young R6/2 HD mice (Tarditi et al., 2006) and knockout of A2aR was found to reverse working memory deficits in young R6/2 mice (Li et al., 2015). Interestingly, A2a receptor inactivation is also linked to improvements in behavioral flexibility and reversal learning (Wei et al., 2011), and so the presence of an early increase in expression, if present in YAC128 mice, could help to account for the observed motor learning deficit.

The presence of these early and transient phenotypes in HD mice suggests that multiple parallel pathophysiological processes may underlie the progression of motor phenotypes in HD. One possibility is that the behavioral changes observed in young HD mice are a direct effect of the huntingtin mutation which is later compensated for during the early disease progression. Alternatively, behavioral phenotypes might be caused by an early compensatory process, and failure of compensation at later stages results in apparent normalization of the behavior. In either case, this suggests that separate and independent processes, as well as eventual neurodegeneration, may cause the slower and progressive development of cognitive and motor phenotypes observed in older (more than four months old) YAC128 mice.

In addition to assessing motor learning, the second primary objective of our study was to investigate the possibility of task-related kinematic abnormalities in YAC128 mice. Mild motor deficits have previously been observed in five- to six-month-old YAC128 mice on the rotarod, horizontal ladder and narrow beam tests (Van Raamsdonk et al., 2005; Di Pardo et al., 2012). However, assessments of skilled motor performance, such as reach-to-grasp and lever-pulling tests, have been infrequently used in genetic models of neurodegenerative disorders. Kinematic analysis of HD models has been primarily focused on gait abnormalities, although these are subtle in the YAC128 model and have only been observed in animals over one year old (Chen et al., 2011). In our task, we found that six-month-old YAC128 animals displayed irregularities in the performance of their trials as compared to WT animals in the second phase of testing. Specifically, many animals were unable to keep the lever at a steady position within the goal range, and progressively released their hold on it over the course of each trial. It seems likely from this behavior that these animals are compensating for a lack of control, and have difficulty maintaining a constant force while holding the lever. This phenotype may be analogous to motor impersistence, a common movement abnormality seen in patients with HD which is characterized by an inability to maintain a constant strength during muscle contractions (Walker, 2007). Motor impersistence is seen in nearly all HD patients, and unlike other primary motor symptoms (such as chorea), it is typically linearly progressive with the disease course (Reilmann et al., 2001). As such, the ability of the cage-system to detect an analogous phenotype in mice is promising for future studies of HD models, and to our knowledge, these results are the first to show such a task-related kinematic abnormality in a mouse model of HD.

In summary, our results further build on the behavioral profile of YAC128 animals at a relatively early phenotypic stage by using an automated and continuously accessible operant motor learning task. These results further validate the use of YAC128 as a model for HD, as we observed several novel phenotypes in these animals that parallel the human disease, including circadian abnormalities and changes in motor behavior on a skilled motor task. This study also provides further evidence for the efficacy of both home-cage assessment, and motor learning tasks for the high-throughput identification of behavioral phenotypes in rodent models of disease.

## Synthesis

Reviewing Editor: Tim Bussey, University of Cambridge Decisions are customarily a result of the Reviewing Editor and the peer reviewers coming together and discussing their recommendations until a consensus is reached. When revisions are invited, a fact-based synthesis statement explaining their decision and outlining what is needed to prepare a revision will be listed below. The following reviewer(s) agreed to reveal their identity: Anthony Hannan, Rebecca Trueman

Both reviewers feel that your work successfully validates a home-cage testing protocol that has utility not only for HD mice, but various other mouse models of nervous system disorders. Each reviewer had a number of comments on the manuscript (listed below), but one major issue to be addressed concerns statistics. In the words of reviewer 2:

“The authors have checked the data to ensure that it doesn't violate the assumptions of an ANOVA, which is excellent practice. However, this has resulted in the need to perform non-parametric tests, which then presents its own set of problems. It has resulted in multiple tests being used on the same data set (for example individual Mann-Whitney or t-tests to compare genotypes at time point, then two Kruskal-Wallis tests to compare the time points across each genotype individually). It is not possible to see if there are interactions between time point and genotype using this method. Also, there are issues with multiple testing. I completely understand that this is a difficult problem to address, but if this approach is taken the authors should be very careful about the wording of the results and discussion, as it is not possible to directly compare differences between WT and YAC128 at one time point to another, you can only infer that an interaction exists. Additionally, if this approach is taken then a correction for multiple comparisons should be considered. The authors may want to consult a statistician to see if there is an alternate method which could be used, for example an alternate form of regression or transformation of the data.”

I think this issue needs to be carefully addressed before the MS would be suitable for publication.

Other comments from the reviewers:

Reviewer 1:

1. The authors state that the animals that did not perform >200 trials or lost >15% body weight were removed and not used further. They should state exactly how many of the HD and WT mice were excluded due to these criteria, at each age.

2. The authors describe 'YAC128-53' in the methods but do not explain this elsewhere (where 'YAC128' is used).

3. In the first sentence of the methods the authors mention maintaining on the FVB background by breeding with wildtype FVB/N animals. Please clarify that FVB/N is correct as the specific sub/strain on which these HD/WT mice were bred.

4. Why were only male mice used, considering that HD occurs in both males and females? It would of interest to know whether sexual dimorphism can be detected on this task, as it has for some other tasks in various HD mouse models.

5. It would helpful to include the key statistics in the results text, instead of only including it all in Table 1. It is unusual to use the superscript letters in the figure legends to refer to statistics in a table. This needs to be made more explicit, and the most important statistical findings more accessible to the reader. At the very least, perhaps each relevant figure legend could state something like 'the superscript letters refer to statistical findings detailed in Table 1'.

6. In Figure 2B, the D1 columns need error bars.

7. On page 10, Fig3B, the authors claimed there was a trend, but the statistics in the table seem to suggest otherwise.

8. The fact that the authors found a behavioral abnormality at 2 months that was not seen in the 4 or 6 month old cohorts is surprising. It is possible that rather than being an unexpected change in phenotype over time, it may be due to variance between cohorts (associated with environmental/epigenetic variables, assuming there was no genetic drift). I.e. it is possible that if 3 independent 2 month-old cohorts had been tested (a couple of months apart each) then only one of them may have shown significant differences. The authors discuss the possibilities explaining the paradoxical phenotypic change at 2 months that is not seen at 4 or 6 months in this progressive neurodegenerative model, however the possibility that this finding reflects inter-cohort variability is also worth briefly discussing.

9. Furthermore, I think known affective and cognitive changes which occur early in this HD mouse model should be discussed in more detail, as they may have impacted on performance in this home-cage assay (including the paradoxical differences at 2, 4 and 6 months of age).

Reviewer 2

1) In the introduction - Line 60 page 3. This is a little misleading as Brooks et al 2012a did not test the mice before 4 months of age. So the deficits may have been present from 2 months.

2) Could it be made clearer how many mice were excluded, at what point and why? For example include a table of these data.

3) Was the person analyzing the data blinded?

4) Page 10 line 205: “Although no overall genotype or age effects were seen, there was a trend in the YAC128 animals towards performing less trials with increasing age (Fig. 2C).” This is a little tenuous - the WT animals performance also decreased.

5) How were the kinematic measures selected from each mouse for analysis? Were measures in place to prevent bias?

6) Were the YAC mice the same weight as the WT? If not, could this explain changes in the kinematic measures?

7) Page 18 - line 385 “Interestingly, 4-month-old YAC128 mice were the only group to show an increase in average lever speed over WT animals in their Phase 2 trials.” Examining figure 5G shows that the WT mice appear to be getting slower over time and the YAC128 mouse performance looks similar across all time points. So this isn't related to hyperkinetic changes in YAC128s which reduce with age. Please alter the discussion to reflect this.
